# Transdermal Rotigotine Improves Sleep Fragmentation in Parkinson's Disease: Results of the Multicenter, Prospective SLEEP-FRAM Study

**DOI:** 10.1155/2015/131508

**Published:** 2015-02-22

**Authors:** Javier Pagonabarraga, Gerard Piñol, Adriana Cardozo, Pilar Sanz, Víctor Puente, Pilar Otermín, Inés Legarda, Tania Delgado, Carmen Serrano, Ernest Balaguer, María Aguirregomozcorta, Ramiro Álvarez, Jaime J. Kulisevsky

**Affiliations:** ^1^Hospital de la Santa Creu i Sant Pau, 08041 Barcelona, Spain; ^2^Hospital Santa Maria de Lleida, 25007 Lleida, Spain; ^3^Hospital de Mataró, 08302 Barcelona, Spain; ^4^Hospital del Mar, 08003 Barcelona, Spain; ^5^Hospital de Granollers, 08401 Barcelona, Spain; ^6^Hospital Son Espases, 07120 Barcelona, Spain; ^7^Corporació Sanitària Parc Taulí, 08208 Barcelona, Spain; ^8^Hospital de Martorell, 08760 Martorell, Spain; ^9^Hospital General de Catalunya, 08915 Barcelona, Spain; ^10^Hospital de Figueres, 17600 Girona, Spain; ^11^Hospital Germans Trias i Pujol, 07014 Barcelona, Spain

## Abstract

Sleep disturbances occur frequently in patients with Parkinson's disease (PD). The aim of this study was to investigate the effects of rotigotine on sleep fluctuations in a sample of PD patients with self-reported complaints of nocturnal awakenings. This prospective, open-label, observational, and multicenter study enrolled consecutive outpatients with PD and administered rotigotine (mean dose 8.9 mg/day) for 3 months. The primary endpoint was the change from baseline in sleep fragmentation, assessed using the sleep maintenance subscale score of the Parkinson's Disease Sleep Scale (PDSS). The newly designed Parkinson's Disease Sleep Fragmentation Questionnaire (PD-SFQ) was used to measure other sleep parameters. A total of 62 patients were enrolled (mean age 70.2 years; 66% male). At 3 months, rotigotine significantly improved sleep fragmentation from baseline on the PDSS-2 sleep maintenance subscale (from 3.4 ± 0.9 to 1.9 ± 1.4; *P* < 0.0001). Rotigotine also significantly improved nocturnal motor symptoms (*P* < 0.0001), restless legs-like symptoms (*P* < 0.005), and nocturia (*P* = 0.004). Rotigotine significantly improved self-reported complaints of sleep fragmentation in PD patients and could be a useful treatment to improve this specific sleep problem in PD. However, these results are based on a small and clinically heterogeneous sample so they must be taken cautiously.

## 1. Introduction

Sleep disturbances occur frequently in patients with Parkinson's disease (PD) [1, 2], with up to 70% of patients experiencing disrupted sleep in the early to midstages of the disease [1, 3], increasing up to 90% over time [4]. The diagnosis, characterization, and management of sleep disorders in patients with PD are not only based on their frequency but on the impact they have on quality of life and the prognostic and clinical correlations that different sleep disorders may entail [5]. Overall, the presence and severity of sleep disorders are independent predictors of poorer quality of life [6–8], high distress scores [9], severity of nonmotor PD symptoms [10, 11], and caregiver's burden [12].

The most frequent sleep disorder in PD is sleep fragmentation [13, 14], clinically defined as the presence of recurrent, involuntary, and frequent nocturnal awakenings that interrupt normal sleep maintenance. It is one of the earliest sleep problems to develop, present in 35–50% of patients within the first 5 years of the disease and increasing in frequency to 60–70% after 10 years of follow-up [4, 13, 14].

Sleep fragmentation in PD has many etiologies including nocturnal recurrence of PD symptoms, coexistent sleep apnea, and nocturia [2, 4]. No specific treatment for sleep fragmentation is currently established in clinical guidelines, but some of its causes could be treated by the use of long-acting dopaminergic agents. Different strategies that provide a more continuous dopaminergic stimulation, such as the use of nocturnal apomorphine [15, 16] or levodopa/carbidopa intestinal gel infusion [17], have been shown to improve sleep by significant reductions of motor PD symptoms at night [18].

Rotigotine, a nonergolinic dopamine receptor agonist, is formulated as a once-daily transdermal delivery system to provide stable plasma drug levels over 24 hours. In the RECOVER study, the rotigotine transdermal patch showed significant improvements in nocturnal motor impairment and global sleep quality, but no specific improvements in sleep maintenance or nocturia were found [19]. As such, the aim of this study was to assess the efficacy of rotigotine on sleep fragmentation in a sample of PD patients with self-reported complaints of unsatisfactory nocturnal awakenings. To describe in greater detail the different sleep problems that may influence sleep fragmentation, the Parkinson's Disease Sleep Fragmentation Questionnaire (PD-SFQ) was designed, which has a wider score range (0–24 points) than the traditional Parkinson's Disease Sleep Scale (PDSS-2) and comprises items that measure awakenings per night, nocturia, restless legs-like symptoms, and nocturnal akinesia. Furthermore, this study aimed to explore the relationships of sleep fragmentation—as measured by the PDSS-2 sleep maintenance subscore—with different nocturnal sleep disturbances and the response of different nocturnal disturbances to rotigotine.

## 2. Methods

### 2.1. Study Design and Patients

This prospective, open-label, observational, and multicenter study enrolled consecutive outpatients fulfilling research diagnostic criteria of PD [20] from centers in Spain with hospital-based neurologists experienced in the treatment of movement disorders. Patients were recruited over a 6-month period and were followed up for 3 months. Patients were eligible if they had self-reported complaints of unsatisfactory nocturnal awakenings or sleep interruption (determined by subject interview) in the previous 4 weeks and if a dopamine agonist was considered the best therapeutic option to improve early-morning, daytime, or nocturnal PD motor symptoms, as determined by the investigator. Patients' disease onset and medication history (including levodopa daily dose, dopamine agonist-levodopa equivalent daily dose (DA-LEDD), and total LEDD) were collected [21]. Patients had to be on stable doses of drugs in the previous 4 weeks. Patients could be receiving dopamine agonists other than rotigotine, either as monotherapy or in combination with levodopa, monoamine oxidase B inhibitors (selegiline, rasagiline), catechol-O-methyltransferase inhibitors, or amantadine. When necessary, overnight switch from oral dopamine agonists to transdermal rotigotine patch was performed [22], at equivalent doses according to previously suggested conversion formulae [21, 23]. During the study, no changes in the doses of other PD medications were allowed.

Individuals were excluded if there was suspicion of atypical or secondary parkinsonism, major psychiatric disorders, or other conditions known to impair mental status other than PD, noncompensated systemic diseases (i.e., diabetes, hypertension, and cancer), or history of skin hypersensitivity to adhesives or other transdermal therapies.

The study protocol was reviewed and approved by the Research Ethics Committee of the Hospital de la Santa Creu i Sant Pau and by the institutional review boards of each participating center. Written informed consent was obtained from all participants. A doctor or second party was present to assess each patient's capacity to consent.

### 2.2. Treatment Outcomes and Procedures

The effect of rotigotine on sleep fragmentation was assessed using the sleep maintenance subscale score of the PDSS-2 [24] at baseline and at 3 months. Motor symptoms were assessed using the Unified Parkinson's Disease Rating Scale III (UPDRS-III) [25] and depressive symptoms by the Geriatric Depression Scale (GDS-15) [26, 27]. Using the PDSS-2, global sleep quality and various aspects of nocturnal sleep problems in PD were also assessed and quantified, as described below [28].

A newly designed questionnaire, the PD-SFQ, was also used to describe different sleep problems that may relate to sleep fragmentation in more detail. While the PDSS-2 measures sleep maintenance and nocturia by the number of days a week each symptom appears, the PD-SFQ measures how many times during the night each sleep disturbance (awakenings, nocturia, restless legs-like symptoms, and nocturnal rigidity) occurs (see Supplementary Material available online at http://dx.doi.org/10.1155/2015/131508).

Enrolled patients received daily doses of rotigotine for 3 months and were instructed to attend a baseline and follow-up visit after giving written informed consent. At 1 month, patients were phoned by the responsible investigator to discuss if any adverse events had occurred and to assess motor function status. A pragmatic dose titration regimen was conducted by a supervising physician at this time if required. At baseline and at 3 months, investigators administered the UPDRS-III, GDS-15, PDSS-2, and PD-SFQ. Total PDSS-2 scores were used to measure global sleep quality and separate PDSS-2 subscale scores were used to assess some of the different sleep problems covered by the scale: sleep efficiency (items 1 and 14), sleep maintenance (item 3), restless legs-like symptoms (items 4 and 5), nocturnal motor symptoms (items 9–13), and nocturia (item 8).

### 2.3. Statistical Analysis

The PDSS-2 sleep maintenance subscale score measured at baseline and 3 months was predefined as the main outcome measure of the study. Secondary outcome variables included changes in the scores on the PD-SFQ and PDSS-2 subscales. To measure significant changes over time, Wilcoxon signed-rank tests were used for items with narrow score range (0 to 5) and paired-sample *t*-tests were used for scores with a wider range. Mean score changes from baseline to the final visit were used for bivariate correlations. A repeated-measures general linear model analysis of variance (GLM ANOVA) was used to build a statistical model to explore variables affected by various factors and interactions between factors. Finally, to determine which sleep symptoms independently predicted improvements in global sleep quality and sleep fragmentation, multiple stepwise regression analyses were performed using the PDSS-2 total score and PD-SFQ total score as dependent variables; those variables that showed a significant relationship in the univariate analysis were treated as independent variables. Data are expressed as means ± standard deviation (SD) for the continuous variables, as percentages for the categorical variables, and as mean ± SD (range) for the ordinal variables. Significance was set at *P* < 0.05. All analyses were performed using SPSS 19.0 (SPSS, Chicago, IL) statistical software.

## 3. Results

In total, 66 patients were screened and 62 patients with PD and sleep fragmentation were enrolled (mean age 70.2 years; 66% male; Table 1). The four patients who were screened and not included in the patient population did not provide written informed consent and refused enrolment in the study. At baseline, patients were receiving a mean dose of rotigotine of 8.5 ± 3.0 mg/day. Before starting rotigotine treatment, 34 patients (54%) were receiving PD treatment with levodopa and a dopamine agonist, 12 (19.4%) were on levodopa monotherapy, 8 (12.9%) were on dopamine agonist monotherapy, and 8 (12.9%) were drug-naïve. All patients who previously received dopamine agonists were receiving extended release formulations. Five patients were receiving entacapone and there was no difference in the number of patients receiving rasagiline between treatment groups.

At the end of the study, the mean dose of rotigotine was 8.9 ± 3.1 mg/day. Two patients dropped out of the study due to nausea and dizziness.

**Figure 1:**
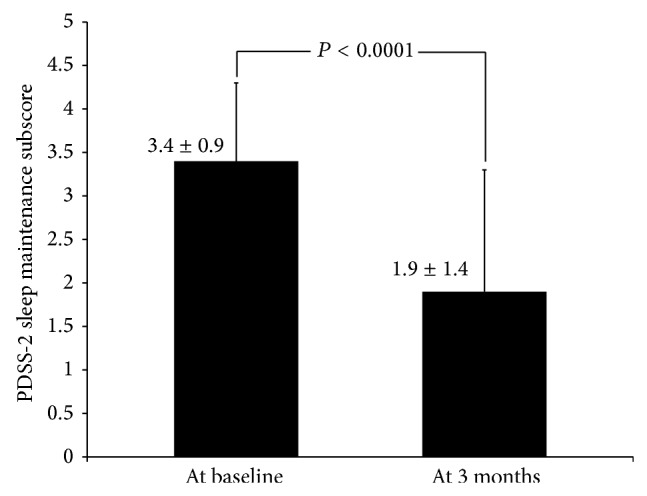
Change from baseline in Parkinson's Disease Sleep Scale (PDSS) sleep maintenance subscale score after 3 months of rotigotine treatment.

After 3 months of treatment, rotigotine significantly improved sleep fragmentation on the PDSS-2 sleep maintenance subscale score (Figure 1). Total PDSS-2 (from 24.3 ± 9.7 to 14.7 ± 8.4; *P* < 0.0001) and UPDRS-III scores (from 23.8 ± 10.7 to 18.4 ± 8.8; *P* < 0.001) were also significantly improved and GDS-15 scores were modestly improved (from 4.7 ± 3.4 to 4.1 ± 3.4; *P* < 0.043) after 3 months of rotigotine treatment. No significant interaction between improvement in the PDSS sleep maintenance subscale scores and 3-month changes in UPDRS-III (*P* = 0.21) or baseline Hoehn and Yahr (*P* = 0.91) and disease duration (*P* = 0.22) was observed.

In the specific sleep disturbances measured by the PDSS-2, rotigotine also significantly improved nocturnal motor symptoms (*P* < 0.0001), restless legs-like symptoms (*P* < 0.005), and nocturia (*P* = 0.004) from baseline (Table 2).

By using mean changes after the initiation of rotigotine, bivariate correlations showed significant improvement in global sleep quality (measured by PDSS-2 total score) correlated with mean score decreases in nocturnal motor symptoms (*r* = 0.80; *P* < 0.001), restless legs-like symptoms (*r* = 0.73; *P* < 0.001), and the PDSS-2 sleep maintenance subscale score (*r* = 0.56; *P* < 0.001). Changes in GDS-15 scores also contributed to better sleep quality, although being less significant (*r* = 0.33; *P* = 0.01).

More specifically, improvement in sleep fragmentation (measured by the PDSS-2 sleep maintenance subscale) significantly correlated with improvement in nocturnal motor symptoms (*r* = 0.34; *P* = 0.008) and restless legs-like symptoms (*r* = 0.27; *P* = 0.02), while changes in GDS-15 (*P* = 0.28) and UPDRS-III (*P* = 0.26) had no effect on sleep fragmentation.

In multiple stepwise regression analyses, the change in PDSS-2 total score with rotigotine at 3 months was independently associated with the improvement in nocturnal motor symptoms (CC = 0.53; *P* < 0.001), restless legs-like symptoms (CC = 0.30; *P* < 0.001), and PDSS-2 sleep maintenance subscale score (CC = 0.30; *P* < 0.001), indicating that the observed improvement in sleep maintenance score contributed independently to better sleep efficiency. The PDSS-2 sleep maintenance subscale score was also independently associated with improvements in restless legs-like symptoms (CC = 0.37; *P* = 0.02) and to a lesser extent with nocturnal motor symptoms (CC = 0.22; *P* = 0.04).

When analyzing the effect of rotigotine in patients with no previous dopamine agonist treatment and in patients switching from one dopamine agonist to rotigotine, all differences remained equally significant. In dopamine agonist naïve patients, minimal interaction between PDSS sleep maintenance improvement (*P* = 0.003) and 3-month change in UPDRS-III (*P* = 0.09) was observed and no interaction with baseline Hoehn and Yahr (*P* = 0.18) or disease duration (*P* = 0.37) occurred. Likewise, GLM ANOVA for repeated measures found significant improvement of PDSS-2 maintenance scores for “de novo” PD patients, patients on levodopa monotherapy, and patients on levodopa-DA combination therapy (Pillai's trace; *P* < 0.001), with no “Time × Treatment group” interaction effect (*P* = 0.692) and no significant differences between groups (Tukey's post hoc tests; *P* > 0.80 for all paired comparisons).

Sleep fragmentation was also significantly improved when measured using the PD-SFQ. After 3 months of treatment, rotigotine improved total PD-SFQ scores from 10.1 ± 3.4 to 6.6 ± 2.7 (*P* < 0.0001). Interestingly, nocturia measured by the PD-SFQ (number of nocturia episodes per night) showed a greater improvement from baseline (from 2.3 ± 1.0 to 1.8 ± 0.8; *P* < 0.0001) than when measured using the PDSS-2 (days with nocturia per week). In addition, 3-month mean changes in PD-SFQ total scores not only correlated with mean changes in nocturnal motor symptoms and restless legs-like symptoms but also correlated with the PD-SFQ nocturia item (*r* = 0.54; *P* < 0.001). The change in PD-SFQ total score was also independently associated with improvement in restless legs-like symptoms (CC = 0.37; *P* = 0.02), PD-SFQ nocturia (CC = 0.35; *P* = 0.01), and nocturnal motor symptoms (CC = 0.22; *P* = 0.04). No interaction between PD-SFQ total score improvement (*P* = 0.001) and 3-month change in UPDRS-III (*P* = 0.60), baseline Hoehn and Yahr (*P* = 0.67), or disease duration (*P* = 0.34) was observed.

Rotigotine was well tolerated. No significant adverse events were reported during the study and no patient stopped the drug due to worsening of motor symptoms or reactions to the transdermal patch.

## 4. Discussion

In the present study, treatment with rotigotine was associated with significant improvements in sleep fragmentation in a prospective sample of PD patients with subjective and spontaneous complaints of sleep interruption. The significant improvement in sleep fragmentation was observed using both the PDSS-2 and a new questionnaire, the PD-SFQ, that assesses specific sleep problems that contribute to the disruption of sleep maintenance in PD. Description and correlational analysis of those sleep disturbances that improved with rotigotine showed that better sleep quality was independently driven by amelioration of nocturnal motor symptoms, restless legs-like symptoms, and sleep fragmentation, whereas alleviation of sleep fragmentation was more specifically driven by improvement in restless legs-like symptoms.

In the double-bind, placebo-controlled trial RECOVER, rotigotine improved sleep efficiency by significant amelioration of the PDSS-2 subscale scores assessing nocturnal and early-morning motor symptoms, restless legs-like symptoms, and pain or muscle cramps [19]. However, in RECOVER sleep maintenance and nocturia did not improve. This is a surprising result and may have repercussions in clinical practice as sleep fragmentation and nocturia are the most frequent sleep disturbances in PD, and they both contribute to a poor quality of life [4, 6, 7]. This negative result could possibly be explained by the recruitment of a heterogeneous sample of PD patients with and without sleep problems and by the noncontrolled distribution of patients with or without sleep fragmentation or nocturia within the two study arms.

To overcome this limitation of the RECOVER study this study aimed to recruit patients with PD complaining of sleep interruption. In this sample, we have observed that restless legs-like symptoms, nocturia, and nocturnal motor symptoms contributed the most to sleep fragmentation. The significant improvement observed in these three clinical domains can be explained by the pharmacokinetic and pharmacodynamic properties of rotigotine, not only in striatal dopamine receptors, but also in the spinal cord, and brainstem nuclei such as medullary raphe nuclei and the pontine micturition center [29].

The long-acting nature of rotigotine on striatal receptors seems the most plausible hypothesis for a better control of Parkinsonian motor symptoms during the night time. Another long-acting dopamine agonist, cabergoline (added to levodopa), has been shown to decrease PDSS-2 scores by improving sleep maintenance and night-time motor symptoms [30], and other therapies providing continuous stimulation of basal ganglia circuits, such as deep brain stimulation [31], nocturnal apomorphine pump [15, 16], and levodopa/carbidopa intestinal gel infusion [17], have also shown specific improvement in sleep fragmentation.

The constant supply of rotigotine over the course of 24 hours and its highest affinity for the D3-receptor [32] may help to explain its efficacy in restless legs-like symptoms. Restless legs syndrome reflects a dysfunction of the dorsoposterior hypothalamic dopaminergic A11 cell group, which modulates spinal cord excitability mainly through D3 receptors [33, 34]. However, given the similar D3 receptor affinity among pramipexole, ropinirole, and rotigotine, we suggest that the higher benefit shown by rotigotine on restless legs-like symptoms may be mostly based on a more continuous dopaminergic stimulation during the night. Additionally, the observed improvements in nocturia could be explained by a better dopaminergic and serotoninergic control exerted by rotigotine on brainstem nuclei. The pontine micturition center is rich in dopamine receptors that inhibit its excitability through projections from the ventral tegmental area [35], and the functionality of the medullary raphe nuclei—that control bladder overactivity and detrusor contractility—could be improved by the specific affinity of rotigotine on 5-hydroxytryptamine 1A receptors [36, 37].

Overall, the selection of patients with self-reported complaints of sleep fragmentation recruited prospectively from daily clinical practice allowed for the observation that rotigotine may improve this specific sleep problem, a finding that was not shown in the RECOVER study. Significant improvement of nocturia after onset of rotigotine and its independent contribution for improving sleep fragmentation were more clearly reflected by assessing this phenomenon with the PD-SFQ, which indicates the number of nocturia episodes per night instead of the number of days per week with nocturia assessed in the PDSS-2. The number of nocturia episodes per night therefore appears to be a clinically and scientifically relevant variable that should be taken into account in future interventional studies. However, it must be noted that the PD-SFQ has not yet been properly validated, and future studies should assess its validity and accuracy.

Some other limitations of our study must be acknowledged. This is an open-label trial, the primary outcome was based on subjective reports, the sample size was not large enough so as to establish firm conclusions, and patients were followed up for only three months. In addition, other sleep problems that can only be assessed using polysomnography, such as sleep disordered breathing, REM sleep behavior disorder, and periodic limb movement syndrome, were not analyzed. However, based on these preliminary results coming from daily clinical practice further research is warranted. A larger sample of patients and a more detailed examination of sleep-related problems contributing to sleep fragmentation using polysomnography or actigraphy would help to verify the potential benefits of rotigotine on different sleep parameters. Finally, given the open-label design of the study, we could not evaluate properly whether the effect of rotigotine on sleep fragmentation was ascribed exclusively to a placebo effect.

## 5. Conclusions

Rotigotine significantly improved self-reported complaints of sleep fragmentation in PD patients and could be a useful treatment to improve this specific sleep problem in PD. The sample in this open-label study is small and clinically heterogeneous, including “de novo” and advanced patients and patients in previous treatment with and without dopamine agonists and, in accordance, the observed results must be taken cautiously.

## Supplementary Material

The Supplementary Material includes the newly designed “Parkinson's Disease – Sleep Fragmentation Questionnaire (PD-SFQ)”. This questionnaire assesses the presence of sleep symptoms that have an influence on sleep fragmentation, such as the number of awakenings per night, nocturia, restless legs-like symptoms, and nocturnal akinesia .

## Figures and Tables

**Table 1:** Demographic and clinical features at baseline.

Characteristic	Rotigotine (*n* = 62)
Age, years	70.2 ± 7 (48–83)
Male, *n* (%)	41 (66.1)
Education, years	9.1 ± 4 (2–20)
Disease duration, years	5.7 ± 4 (0.5–24)
UPDRS-III	23 ± 10 (5–60)
Hoehn and Yahr	2.2 ± 0.8 (1–4)
Total LEDD, mg/day	526 ± 436 (0–1800)
GDS-15	5 ± 4 (0–15)
Total PDSS-2 score	23.5 ± 9 (10–46)
PD-SFQ	10.1 ± 3 (5–20)
Rotigotine dose, mg/day	8.5 ± 3 (4–16)

All values are mean ± standard deviation (range) unless otherwise stated.

GDS-15, Geriatric Depression Scale; LEDD, levodopa equivalent daily dose; PD-SFQ, Parkinson's Disease Sleep Fragmentation Questionnaire; PDSS-2, Parkinson's Disease Sleep Scale; UPDRS, Unified Parkinson's Disease Rating Scale.

**Table 2:** Parkinson's Disease Sleep Scale (PDSS) subscale scores at baseline and 3 months in patients with Parkinson's disease receiving rotigotine.

PDSS subscale score	Baseline	3 months	*P* value
Sleep maintenance	3.4 ± 0.9	1.9 ± 1.4	*P* < 0.0001
Nocturnal motor symptoms	6 ± 5	3 ± 3	*P* < 0.0001
Sleep efficiency	4.3 ± 2	2.3 ± 2	*P* < 0.0001
Restless legs-like symptoms	2.5 ± 2	1.4 ± 2	*P* < 0.005
Nocturia	3.6 ± 0.9	3.0 ± 1.0	*P* < 0.004
