# Supplementary material for: Transdermal Rotigotine Improves Sleep Fragmentation in Parkinson's Disease: Results of the Multicenter, Prospective SLEEP-FRAM Study
Source: Parkinsons Dis. 2015 Feb 22;2015:131508. doi: 10.1155/2015/131508 (PMC4352510; doi:10.1155/2015/131508)
Supplement: Supplementary file 1 — The Supplementary Material includes the newly designed “Parkinson's Disease – Sleep Fragmentation Questionnaire (PD-SFQ)”. This questionnaire assesses the presence of sleep symptoms that have an influence on sleep fragmentation, such as the number of awakenings per night, nocturia, restless legs-like symptoms, and nocturnal akinesia . [file 131508.f1.pdf]

Supplementary material for article

Transdermal rotigotine improves sleep fragmentation in Parkinson's disease: results of the multicentre, prospective SLEEP-FRAM study

Authors: Javier Pagonabarraga[1], Gerard Piñol[2], Adriana Cardozo[2], Pilar Sanz[3], Víctor Puente[4], Pilar Otermín[5], Inés Legarda[6], Tania Delgado[7], Carmen Serrano[8], Ernest Balaguer[9], María Aguirregomozcorta[10], Ramiro Álvarez[11], Jaime Kulisevsky J[1]

Corresponding author: Javier Pagonabarraga

Hospital de la Santa Creu i Sant Pau, C/ Mas Casanovas 90, Barcelona 08041, Spain.

Phone: 935565986 / Fax 935565748; Email: jpagonabarraga@santpau.cat

Journal: Journal of Neurology

Parkinson's Disease – Sleep Fragmentation Questionnaire (PD-SFQ)

(0 – 24 points)

Over the ***last 2*** *weeks*, on average, how often have you been bothered by the following problems?

1. How many times do you wake up during the night?
    a. Never.
    b. Once.
    c. Twice.
    d. Three times.
    e. ≥ 4 times.
2. How long does it take you to fall asleep again after waking up?
    a. Less than 10 minutes.
    b. Between 10 and 30 minutes.
    c. Between 30 and 60 minutes.
    d. Between 60 and 120 minutes.
    e. >120 minutes.

3. How many hours do you actually sleep every night?

    a. > 8 hours.

    b. Between 6 – 8 hours.

    c. Between 4 – 6 hours.

    d. Between 2 – 4 hours.

    e. < 2 hours.

4. How many times must you urinate during the night?

    a. Never.

    b. Once.

    c. Twice.

    d. Three times.

    e. ≥ 4 times.

5. How many times do you wake up at night because of inner restlessness or pain in your legs that improves shortly after you move them or get up and walk around?

    a. Never.

    b. Once.

c. Twice.

d. Three times.

e. ≥ 4 times.

6. How many times do you wake up at night with pain, muscle cramps, or rigidity in your legs that does not improve even if you move them or get up and walk around?

    a. Never.

    b. Once.

    c. Twice.

    d. Three times.

    e. ≥ 4 times.
